# Laparoscopic total pelvic exenteration using transanal minimal invasive surgery technique with en bloc bilateral lymph node dissection for advanced rectal cancer

**DOI:** 10.1186/s40792-016-0198-6

**Published:** 2016-07-26

**Authors:** Kengo Hayashi, Masanori Kotake, Daiki Kakiuchi, Sho Yamada, Masahiro Hada, Yosuke Kato, Chikashi Hiranuma, Kaeko Oyama, Takuo Hara

**Affiliations:** Department of Surgery, Koseiren Takaoka Hospital, 5-10, Eirakucho, Takaoka, Toyama 933-8555 Japan

## Abstract

A 59-year-old man presenting with fecal occult blood visited our hospital. He was diagnosed with advanced lower rectal cancer, which was contiguous with the prostate and the left seminal vesicle. There were no metastatic lesions with lymph nodes or other organs. We performed laparoscopic total pelvic exenteration (LTPE) using transanal minimal invasive surgery technique with bilateral en bloc lateral lymph node dissection for advanced primary rectal cancer after neoadjuvant chemoradiotherapy. The total operative time was 760 min, and the estimated blood loss was 200 ml. LTPE is not well established technically, but it has many advantages including good visibility of the surgical field, less blood loss, and smaller wounds. A laparoscopic approach may be an appropriate choice for treating locally advanced lower rectal cancer, which requires TPE.

## Background

Total pelvic exenteration (TPE) was first described by Brunschwig as a palliative treatment for the terminal stages of advanced pelvic malignancies [1]. Although TPE is highly invasive, it is a potentially curative procedure for locally advanced rectal cancer invading adjacent organs. One drawback of TPE is its high rate of postoperative complications and high morbidity [2, 3]. Recently, the usefulness of laparoscopic extended surgery for rectal cancer was reported, and it can decrease a complication rate [4, 5]. Here, we report our experience of laparoscopic total pelvic exenteration (LTPE) with en bloc lateral lymph node dissection after neoadjuvant chemoradiotherapy for advanced primary rectal cancer.

## Case presentation

A 59-year-old man was admitted to our hospital for the treatment of a rectal tumor.

**Fig. 1:**
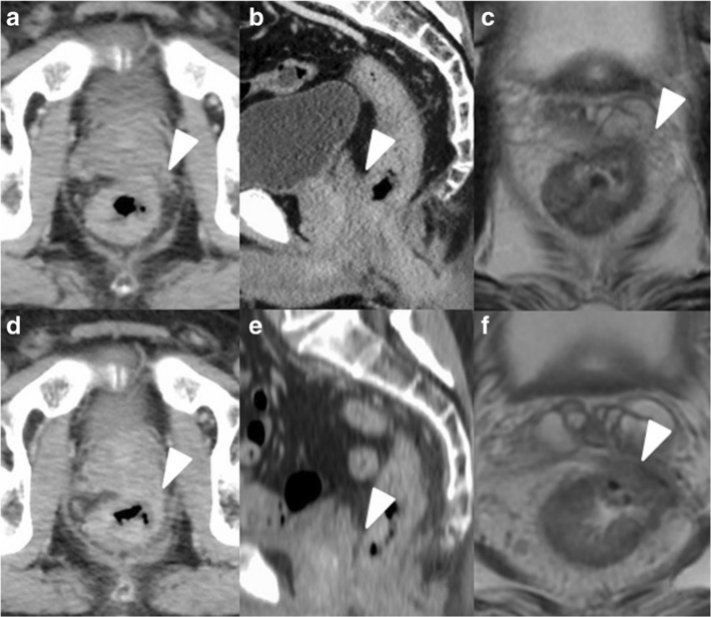
CT and MRI show the tumor invading the left seminal grand and prostate (*white arrowheads*). After CRT, the degree of invasion to adjacent organs was unchanging. **a**–**c** Pre-CRT CT, MRI image. **d**–**f** Post-CRT CT, MRI image

The tumor was found via colonoscopy during an examination at a medical checkup. The colonoscopy revealed an ulcerated tumor at the anterior rectum, 4 cm from the anal verge. Histopathologic examination revealed moderately differentiated adenocarcinoma. CT and MRI scans showed that the rectal tumor was contiguous with the prostate and the left seminal vesicle. PET-CT showed no evidence of metastasis. The carcinoembryonic antigen (CEA) level was elevated at 105.1 ng/mL. After neoadjuvant chemoradiotherapy (CRT) (S-1 100 mg/m2/6 weeks, 2 Gy*25/5 weeks = 50 Gy), CEA level decreased to 24.3 ng/mL but the size of the tumor and the degree of invasion were unchanging (Fig. 1). From the CT and MRI, the invasion to adjacent organs was still undeniable; therefore, we thought that TPE was appropriate surgery for this patient from the point of view of curability. We performed LTPE 8 weeks after chemoradiotherapy.

**Fig. 2:**
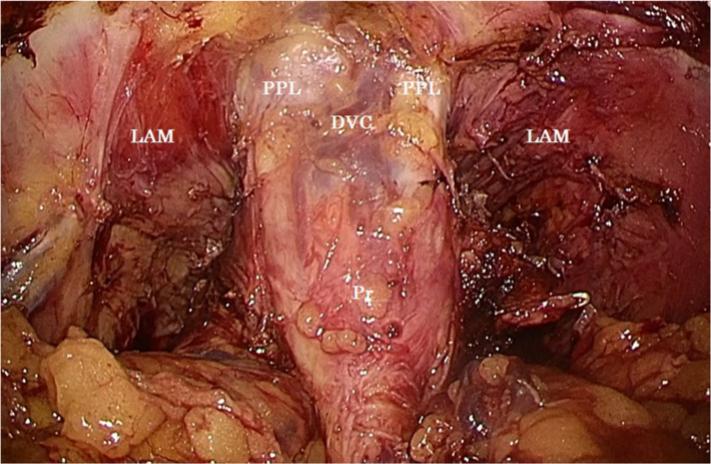
Laparoscopic view before dissecting dorsal vein complex. *DVC* dorsal vein complex, *PPL* puboprostatic ligament, *LAM* levator ani muscle, *Pr* prostate

The patient was placed in the Trendelenburg lithotomy position. The surgeon and the cameraman stood on the patient’s right side. Five ports were placed as follows: 12-mm ports at the umbilicus and the lower right quadrant and 5-mm ports at the upper right and left and lower left quadrant. First, the inferior mesenteric artery and vein were skeletonized, clipped, and divided with adequate lymph node dissection. After dissecting the mesentery of the sigmoid colon, the sigmoid colon was transected with a linear stapler (Endo GIA, purple, 60 mm; Covidien, Norwalk, CT, USA). Next, the left ureter was dissected to the level of the ureterovesical junction, where it was clipped and divided. The surgeon moved to the patient’s left side, and the same procedure was performed for the right ureter after mobilizing the cecum to the level of the duodenum. We did not place ureteral catheters. The superior hypogastric nerve was divided at the level of the aortic bifurcation. The surgeon moved to the patient’s right side again and dissected the posterior rectal space. After recognizing the left internal iliac vessels, the left vas deferens was clipped and divided. The branches of left internal iliac artery were clipped and divided until the entry point to Alcock’s canal. Left en bloc lateral lymph node dissection was performed, and the same procedure was then performed on the right side. Bilateral obturator nerves were preserved. After dissecting the dorsal cavity of the rectum to the level of the levator ani muscle, the prerectal peritoneum was incised and the medial ligament was clipped and dissected. The Retzius space was dissected, and the dorsal vein complex (DVC) was exposed after incising the bilateral puboprostatic ligament and the endopelvic fascia (Fig. 2). At first, we tried to dissect the DVC with a linear stapler (Endo GIA, purple, 60 mm), but we were unsuccessful because of the thickness of the DVC. The black staple, used for thick tissue, was insufficient to dissect it. We dissected the DVC carefully with Ligasure™ Maryland (Covidien), taking care to not cause bleeding of the cutoff stump. The urethra was exposed and dissected with a linear staple (Endo GIA, camel, 45 mm). The levator ani muscle was divided laterally at its origin from the tendinous arch.

**Fig. 3:**
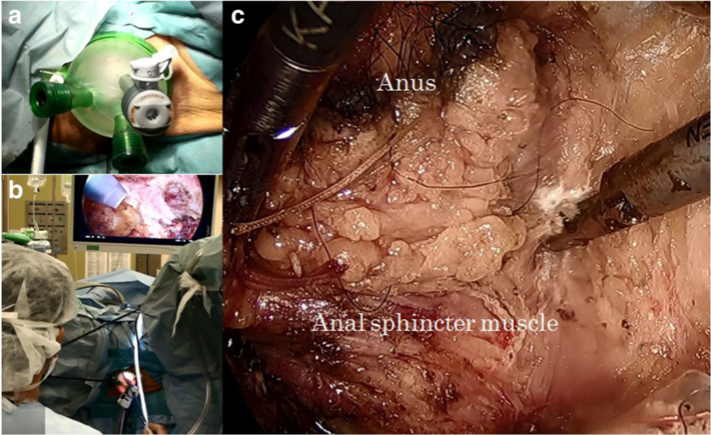
**a** A multiple access port was set for TAMIS. **b**, **c** TAMIS technique helped to maintain a good visual field during the anal-side procedure

**Fig. 4:**
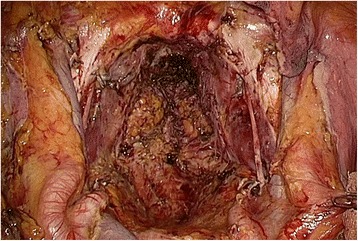
Laparoscopic view after total pelvic exenteration with en bloc lateral lymph node dissection

After the patient’s legs were elevated, the surgeon and the first assistant moved to the anal side and closed the anus with double purse-string suture. After the perianal skin was incised, a multiple access port (GelPoint®Mini; Applied Medical, Rancho Santa Margarita, USA) was set using the transanal minimal invasive surgery (TAMIS) technique. Three small ports were installed, and perirectal tissue and muscles were dissected until we reached the abdominal cavity under the pneumoperirectum (Fig. 3). The entire specimen was removed from the perineal opening, and the perineal opening wound was closed (Fig. 4).

An ileal conduit and a sigmoidostomy were then constructed extracorporeally through the extended umbilical incision. The total operative time was 760 min, and the estimated amount of bleeding was 200 ml. The postoperative course was good, and the patient was discharged on postoperative day 15. Histopathological analysis revealed no apparent invasion of the tumor to adjacent organs, and the pathological stages were ypT3 and ypN0.

### Discussion

Currently, laparoscopic technique is used for various surgeries; thus, opportunity to perform laparoscopic surgery has increased for many surgeons. The laparoscopic approach has advantages not only for patients but also for surgeons, i.e., less pain, smaller wounds, earlier recovery, and a magnified view. LTPE is a challenging and complicated operation, but some studies have reported on the safety and feasibility of it not only for urologic or gynecologic malignancies [6, 7] but also colorectal malignancies [3, 5, 8, 9]. This is our initial experience with LTPE, and so far, we have not encountered any complications. We have routinely performed lateral lymph node dissection for locally advanced lower rectal cancer, and we believe that this previous experience enabled our current success. Upon histopathological analysis, no apparent invasion of the tumor to adjacent organs was observed, but this was difficult to detect before the surgery; therefore, this patient should have been treated by TPE. This case did not include a posterior invasion, so the surgery time was comparatively short. We did not reconstruct the perineal defect, change the patient’s position, or place ureteral catheters after dissecting the ureters. We believe that these factors contributed to shortening the surgery time. We did not monitor the urine volume after dissecting the ureters, but intraoperative and postoperative complications did not occur.

During the anal-side procedure, we used the TAMIS technique. With the TAMIS technique, we could keep a good visual field even after penetration to the abdominal cavity because the pressure of pneumoperirectum can be kept by the pneumoperitoneum. In addition, the perineal wound becomes smaller, which may lead to reduction in the perineal surgical site infection, and pnuemoperirectum can reduce the amount of bleeding. In the middle of the procedure, the wound retractor broke due to contact with the blade of the Sonicision™ (Covidien) and the pneumoperirectum could not be kept thereafter, which will require further attention.

## Conclusions

In conclusion, LTPE is a potentially safe and feasible procedure. However, reports on LTPE for advanced rectal cancer are scarce; therefore, more studies are necessary to evaluate the long-term safety of LTPE.

## Consent

A consent was obtained from the patient for publication and presentation, when we obtained an informed consent for surgery.
